# Gut microbiota regulation of P-glycoprotein in the intestinal epithelium in maintenance of homeostasis

**DOI:** 10.1186/s40168-021-01137-3

**Published:** 2021-09-07

**Authors:** Sage E. Foley, Christine Tuohy, Merran Dunford, Michael J. Grey, Heidi De Luca, Caitlin Cawley, Rose L. Szabady, Ana Maldonado-Contreras, Jean Marie Houghton, Doyle V. Ward, Randall J. Mrsny, Beth A. McCormick

**Affiliations:** 1 Department of Microbiology and Physiological Systems, University of Massachusetts Medical School, Worcester, MA 01605 USA; 2 Program in Microbiome Dynamics, University of Massachusetts Medical School, Worcester, MA 01605 USA; 3 Graduate School of Nursing, University of Massachusetts Medical School, Worcester, MA 01605 USA; 4 Department of Pharmacy and Pharmacology, University of Bath, Bath, BA2 7AY UK; 5 Division of Gastroenterology and Nutrition, Department of Pediatrics, Boston Children’s Hospital, Harvard Medical School, Boston, MA 02115 USA; 6 Ferring Pharmaceuticals, San Diego, CA 92121 USA; 7 Division of Gastroenterology, Department of Medicine, University of Massachusetts Medical School, Worcester, MA 01605 USA

**Keywords:** P-glycoprotein, Multi-drug resistance transporter, Endocannabinoid, Inflammatory bowel diseases, Ulcerative colitis, Inflammation, Intestinal epithelium, Short-chain fatty acids, Secondary bile acids, Microbiome

## Abstract

**Background:**

P-glycoprotein (P-gp) plays a critical role in protection of the intestinal epithelia by mediating efflux of drugs/xenobiotics from the intestinal mucosa into the gut lumen. Recent studies bring to light that P-gp also confers a critical link in communication between intestinal mucosal barrier function and the innate immune system. Yet, despite knowledge for over 10 years that P-gp plays a central role in gastrointestinal homeostasis, the precise molecular mechanism that controls its functional expression and regulation remains unclear. Here, we assessed how the intestinal microbiome drives P-gp expression and function.

**Results:**

We have identified a “functional core” microbiome of the intestinal gut community, specifically genera within the *Clostridia* and *Bacilli* classes, that is necessary and sufficient for P-gp induction in the intestinal epithelium in mouse models. Metagenomic analysis of this core microbial community revealed that short-chain fatty acid and secondary bile acid production positively associate with P-gp expression. We have further shown these two classes of microbiota-derived metabolites synergistically upregulate P-gp expression and function in vitro and in vivo. Moreover, in patients suffering from ulcerative colitis (UC), we find diminished P-gp expression coupled to the reduction of epithelial-derived anti-inflammatory endocannabinoids and luminal content (e.g., microbes or their metabolites) with a reduced capability to induce P-gp expression.

**Conclusion:**

Overall, by means of both in vitro and in vivo studies as well as human subject sample analysis, we identify a mechanistic link between cooperative functional outputs of the complex microbial community and modulation of P-gp, an epithelial component, that functions to suppress overactive inflammation to maintain intestinal homeostasis. Hence, our data support a new cross-talk paradigm in microbiome regulation of mucosal inflammation.

**Video abstract**

**Supplementary Information:**

The online version contains supplementary material available at 10.1186/s40168-021-01137-3.

## Introduction

The human intestine is home to a continuous balancing act between the host immune response, a large community of resident bacteria, and a single-cell epithelial layer that functions to maintain a barrier between them. Disruptions in this fine balance can lead to chronic and acute intestinal inflammation, which is a significant cause of morbidity and mortality worldwide. In particular, neutrophils play a critical role in actively driving disease with a high density of neutrophils being present in the large epithelial wounds that represent precursors of ulcerated lesions characteristic of inflammatory bowel disease (IBD). Understanding the mechanisms that drive neutrophil infiltration during disease is therefore of critical importance to public health. We previously discovered two ATP-binding cassette (ABC) transporter systems, multi-drug resistance protein 2 (MRP2) and P-glycoprotein (P-gp), that act in opposition at the epithelial surface to maintain intestinal homeostasis through regulation of neutrophil migration [1, 2]. MRP2 drives the critical step of neutrophil migration across the epithelial barrier (i.e., transepithelial migration) by apical release of the potent chemoattractant hepoxilin A_3_ (HXA_3_) [1]. Under homeostatic conditions, P-gp-mediated epithelial export of N-acyl ethanolamine-type (NAE) endocannabinoids (eCB) suppresses this neutrophil transepithelial migration through eCB engagement with the neutrophilic cannabinoid receptor 2 (CB2) [2]. Therefore, these ABC transporters and their endogenous substrates play an underappreciated yet fundamental role in balancing immune-modulation at the intestinal mucosal surface.

These observations also underscore the importance of intestinal epithelial cells in balancing, through coordinating the P-gp/MRP2 axis, constitutive secretion of anti-inflammatory lipids with the stimulated secretion of pro-inflammatory lipids to appropriately control neutrophil infiltration while remaining poised to trigger a protective immune response. In the absence of disease, P-gp is highly expressed on the surface of intestinal epithelial cells and serves a crucial role in promoting intestinal barrier health both by limiting HXA_3_-driven neutrophil infiltration and through the export of toxins and xenobiotics [2, 3]. The importance of maintaining P-gp expression is evidenced by the association of functional P-gp deficiency with colonic inflammation: single nucleotide polymorphisms affecting P-gp expression or function in humans are linked to IBD [4, 5], and, further, P-gp (*mdr1a*) knockout mice develop spontaneous colitis that mimics human ulcerative colitis (UC) [6–8]. The P-gp/MRP2 axis shifts with intestinal inflammation, including in UC patients, with a reduction in P-gp expression and an induction of MRP2 expression [1, 9, 10].

Coinciding with this imbalance, UC is associated with perturbation of the intestinal microbiota, including an expansion of *Proteobacteria* and a reduction of *Firmicutes* bacteria including members of the *Clostridia* and *Bacilli* classes, many of which have potential to produce short-chain fatty acids and secondary bile acids [11–14]. Specifically, UC patients have a reduced abundance of butyrate-producing bacteria such as *Roseburia hominis* and *Faecalibacterium prausnitzii*, as well as reduced concentration of short-chain fatty acids and secondary bile acids in the intestine [14–16]. However, the cause versus effect of this microbial dysbiosis in UC is unclear. Moreover, the impact of this dysbiosis on the regulation of the P-gp/MRP2 axis in the intestinal epithelium is unknown.

Here we utilize in vivo models of microbiota perturbation including antibiotic treatment and stratification, as well as reconstitution of germ-free mice, to examine the role of the intestinal microbiota in regulating the P-gp axis in the intestinal epithelium. We performed metagenomic sequencing to identify the bacterial community that is required and sufficient for P-gp induction in vivo. Through reductionist approaches, we show that short-chain fatty acids and secondary bile acids act in concert to induce functional P-gp in vitro. Additionally, through collection of intestinal samples from a cohort of UC patients, we find reduced P-gp expression and function compared to healthy control subjects that is driven by the luminal compartment. These studies identify a mechanistic link between the commensal microbiota in driving functional P-gp expression that is critical to maintain intestinal homeostasis through suppression of neutrophil transmigration.

## Methods

### Specific pathogen-free (SPF) mice

Female C57BL/6J wild-type mice were purchased from Jackson Laboratories (Bar Harbor, ME) and housed 3–5 mice per cage, provided with irradiated standard chow (Prolab IsoPro #5P76) and acidified water ad libitum. Animals were used at age 8–12 weeks after 4-week acclimation to housing facility. All experiments involving SPF mice were performed in accordance with the University of Massachusetts Medical School IACUC (Protocol A-1993-17). Female mice were used in this study for antibiotic treatment and cecal microbiota transfer to maintain consistency with prior studies [2, 17], as well as to enable mixed co-housing of animals. Moreover, the disease we are modeling, colitis, is well-documented to have no sex predominance [18–20].

### Germ-free mice

All studies with germ-free mice were carried out with approval by Boston Children’s Hospital Institutional Animal Care and Use Committee. Male and female WT C57BL/6J mice derived under germ-free conditions were maintained in sterile isolators with autoclaved food, water, and bedding.

### Antibiotic treatment of SPF mice

Female WT SPF mice were treated for 1–14 days with non-acidified drinking water containing ampicillin (0.5 g/L; Fisher), vancomycin (0.25 g/L; Sigma), neomycin (0.25 g/L; Sigma), and/or metronidazole (0.5 g/L; Sigma) for AVNM treatment, modeled after [21], or for 7 days with non-acidified drinking water containing streptomycin (5 mg/mL; Sigma) or cefoperazone (0.5 mg/mL; Sigma). Non-acidified drinking water was used as an untreated control. Water was changed every 3–4 days. Following euthanization, colons were excised, rinsed in HBSS, and flash-frozen as 0.5 cm pieces for further analysis of protein expression.

### Colonization of germ-free mice

Colonization of germ-free mice was modeled after [22–25]. A cecal homogenate was prepared from a C57BL/6J donor mouse obtained from Jackson Laboratories (Bar Harbor, ME) by harvesting cecal contents, transferring to 2 mL sterile PBS in a tissue grinder, and homogenizing for 60 s. Contents were centrifuged for 3 min at 500×*g* to pellet large insoluble material. Six-week-old male and female recipient germ-free mice were colonized by one-time oral gavage with 150 mL of freshly prepared cecal homogenate under sterile conditions. Colonized mice were housed in sterile cages with autoclaved food, water, and bedding for 14 days when colon tissue was harvested, flushed with PBS, and flash frozen for protein analysis. Control mice were maintained in sterile isolators and colon tissue was harvested at 8 weeks of age.

### Butyrate delivery to germ-free mice

Sodium butyrate (Alfa Aesar) was dissolved in autoclaved drinking water at 100 mM final concentration and sterile filtered. Six-week-old female and male germ-free C57BL/6J mice were given regular drinking water or sodium butyrate drinking water for 14 days. In studies with conventionally raised mice and germ-free mice, there was no difference in water intake for mice given regular water compared to water supplemented with sodium butyrate. After 14 days, mice were euthanized, colon tissue was isolated, flushed with PBS, and flash frozen for protein analysis.

### Cholestyramine bile acid sequestration

Powdered irradiated standard mouse chow was mixed with 2% w/w cholestyramine resin (Sigma), modeled after [26]. Powdered chow mixture was reformed into pellets with autoclaved water, air dried, and provided to wild-type SPF mouse in regular cages as the sole food source for 14 days. Chow pellets reformed from powdered chow without cholestyramine served as the control. On day 14, mice were euthanized, and intestines were excised. Colons were rinsed in HBSS and flushed with PBS, while ceca were cut open and contents thoroughly emptied into UltraPure H_2_O (Gibco). Colon and ceca were cut into small pieces (0.5 cm) and flash frozen for further analysis.

### Stool sample collection and DNA extraction

All mouse stool samples were collected immediately upon voiding into sterile vials and flash-frozen in liquid nitrogen, with subsequent storage at – 80 °C. For microbiome analyses, DNA was extracted from fecal samples using the DNeasy PowerSoil Kit (Qiagen). DNA was stored in 1× TE at – 20 °C until further processing.

### 16S qPCR

Quantitative real-time PCR (qPCR) was performed using iQ SYBR Green Supermix (BioRad) and Step One Plus real-time PCR system (Applied Biosystems). The amplification program was set to an initial denaturation step at 95 °C for 3 min, followed by 40 cycles of 15 s denaturation at 95 °C and 1 min annealing/extension at 60 °C. The following primer sets were used: 16S V3-V4 universal primers, 5′-ACTCCTACGGGAGGCAGCAG-3′ and 5′-GGACTACHVGGGTWTCTAAT-3′; mouse GAPDH, 5′-TGGCAAAGTGGAGATTGTTGCC-3′ and 5′-AAGATGGTGATGGGCTTCCCG-3′, purchased from IDT. Relative amount of 16S DNA was related to mouse genomic DNA for each sample (ΔC_t_) as described in [27], prior to calculation of ΔΔC_t_ relative to the control group in order to calculate fold difference in 16S.

### Whole genome sequencing and processing

Sequencing libraries were constructed using the Nextera XT DNA library prep kit (Illumina) and sequenced using the NextSeq® 500/550 High Output Kit v2 with 150 bp paired-end reads. Reads were first trimmed and filtered using Trimmomatic and Bowtie2 as part of the KneadData pipeline. Microbial communities were then profiled using metaphlan2. Pathway and gene family abundance were profiled using Humann2. Gene family abundance was then regrouped to Kegg Orthology (KO) groups before normalization to counts per million (cpm). Normalized data were then used for downstream statistical analysis in R, including Phyloseq, ggplot, vegan, and EnhancedVolcano packages.

### Preparation of intestinal tissue and LC/MS/MS analysis of bile acids

The internal standard (IS) solution was prepared in 50% methanol at D_4_-CA 20 μM, D_4_-DCA 20 μM, D_4_-CDCA 5 μM, D_4_-UDCA 2 μM, and D_4_-LCA 2 μM.

To prepare the tissue samples, 20 mg of tissue powder (ground in liquid nitrogen, stored in – 80 °C) was mixed with 200 μL of 50% methanol and homogenized. Fifty-five microliters of tissue homogenate was mixed with 5 μL of IS solution and 400 μL of ice-cold acetonitrile, vortexed for 30 s and centrifuge at 16,000×*g* for 10 min. The supernatant was dried under nitrogen gas, re-suspended in 60 μL of 50% methanol and injected for analysis.

A mixed bile acid standard was prepared in 50% methanol at CA 320 μM, DCA 640 μM, CDCA 80 μM, UDCA 80 μM, and LCA 160 μM, and then serially diluted for preparing the standard solutions. To obtain the standard curve samples, 5 μL of each standard solution was mixed with 5 μL of IS solution, 5 μL of PBS solution, 45 μL of 50% methanol and 400 μL of ice-cold acetonitrile, votexed for 30 s and centrifuge at 16,000×*g* for 10 min. The supernatant was dried under nitrogen gas, re-suspended in 60 μL of 50% methanol and injected for analysis.

Five microliters of samples were injected into a Thermo Scientific Ultimate 3000 HPLC system coupled with a Thermo Scientific TSQ Quantiva triple quadrupole mass spectrometer. The chromatographic separation was performed on a Waters Acquity BEH C18 column (1.7 μm, 2.1 × 100 mm). The column oven temperature was maintained at 25 °C. The mobile phase consisted of water containing 10 mM ammonium acetate (A) and acetonitrile (B) and pumped at a flow rate of 0.25 mL/min. The gradient conditions were set as follows: 0 min, 25% B; 2.5 min, 25% B; 4.0 min, 27% B; 5.0 min, 28% B; 6.5 min 28% B; 7.0 min 29% B; 8.0 min 32% B; 9.0 min 36% B; 10.0 min 36% B; 11.0 min 37% B; 12.0 min 41% B; 13.0 min 46% B; 14.5 min 55% B; 15.5 min 67%; 16.0 min 75% B; 16.5 min 98% B; 20.0 min 98% B; 20.2 min 25% B; 25.0 min 25% B; giving a totally 25-min run.

Ionization was operated in the negative mode (voltage, 2.8 kV). The parameters was set as follow: sheath gas, 35 Arb, aux gas, 15 Arb, vaporizer temperature, 250 °C, ion transfer tube temperature, 325 °C. Multiple reaction monitoring (MRM) was performed using a cycle time of 0.4 s, CID gas pressure of 1.5 mTorr, Q1 resolution (FWHM) of 0.7 and Q3 resolution (FWHM) of 0.7. The retention time (RT) and MRM transitions used for quantitation are as below. Each bile acid was quantified based on the peak area ratio to its isotope-labeled standard.

**Table:** 

Bile acid	RT (min)	MRM transition	Collision energy (V)
CA	10.4	407.3 > 407.3	10.25
UDCA	10.2	391.3 > 391.3	10.25
CDCA	13.2	391.3 > 391.3	10.25
DCA	13.6	391.3 > 391.3	10.25
LCA	16.5	375.3 > 375.3	12.20
D_4_-CA	10.4	411.3 > 411.3	10.25
D_4_-UDCA	10.2	395.3 > 395.3	10.25
D_4_-CDCA	13.2	395.3 > 395.3	10.25
D_4_-DCA	13.6	395.3 > 395.3	10.25
D_4_-LCA	16.5	375.3 > 375.3	12.20

### Human cell lines

H292 lung epithelial carcinoma cells (CRL 18-48; ATCC) were grown in RPMI 1640 with 10% heat-inactivated FBS. T84 intestinal epithelial cells at passages 50 to 79 (ATCC) were grown in growth media [a 1:1 mixture of Dulbecco’s modified Eagle’s medium (DMEM) and Ham’s F-12 nutrient mixture (Thermo Fisher Scientific) supplemented with 14 mM NaHCO_3_, 15 mM HEPES buffer, 100 units/mL penicillin/streptomycin, and 7.5% heat-inactivated fetal bovine serum (FBS, Hyclone)]. Cells were maintained at 37 °C and 5% CO_2_. Monolayers were grown on collagen-coated tissue culture-treated 12-well plates, 6-well Transwell® plates, 24-well Transwell® plates, or 96-well Transwell® plates (Costar) and used 6 to 8 days after plating. Prior to incubation with fecal supernatants, cells were serum starved for 1 h in serum-free T84 growth medium. For metabolite incubations, sodium butyrate (Sigma), sodium propionate (Sigma), sodium acetate (Sigma), lithocholic acid (Cayman Chemicals), deoxycholic acid (Sigma), and/or ursodeoxycholic acid (Sigma) in 0.5% DMSO in growth media was added to the apical side of cells and incubated for 24 h. For antibiotic incubation, T84 cells were incubated with 0.5 g/L ampicillin, 0.25 g/L vancomycin, 0.25 g/L neomycin, and/or 0.5 g/L metronidazole in growth media for 24 h before collection of lysates.

### Rhodamine retention assay

Cells incubated with or without metabolites, as described above, were washed with Hank’s buffered saline solution (HBSS, Gibco). After a 30-min preincubation of cells with 60 nM PSC833/Valspodar (Sigma) in 0.5% DMSO, cells were then incubated for 1 h in 10 μM Rhodamine123 (Sigma) in 0.5% DMSO in HBSS. Cells were washed with HBSS and incubated 1 h to allow efflux of Rho123, similar to that previously described [28]. Cells were washed with phosphate-buffered saline (PBS) and then lifted with 0.25% trypsin-EDTA (Gibco) for 15 min at 37 °C. Cells were washed and set to 0.5 × 10^6^ cells/mL/sample in cold 1× stain buffer (PBS, 3% FBS, 1 mM EDTA). Cells were washed in 1× stain buffer and resuspended in 4=, 6-diamidino-2-phenylindole (DAPI; Thermo) for live/dead differentiation. Cell suspensions were filtered through 40-μm nylon mesh prior to data collection on a MACSQuant10 flow cytometer (Miltenyi Biotec). Data were analyzed using the FlowJo software (TreeStar). The geometric mean of Rho123 (FITC channel) of the DAPI- population was computed for each sample and normalized to an untreated medium control or DMSO control sample.

### Production of enriched HXA_3_

HXA_3_ was enriched from *Pseudomonas aeruginosa* infection of H292 cells as previously described [2]. Briefly, *P. aeruginosa* were grown as shaking culture overnight in Luria broth (LB) at 37 °C for 14–16 h. Cultures were washed once in HBSS and resuspended at 8 × 10^9^ CFU/mL. H292 cells at confluency in T175 flasks were infected with 300μL of this bacterial suspension (2–3 × 10^9^ CFU) in HBSS for 1 h. H292 cells were washed twice and incubated in HBSS alone for 2 h. HBSS supernatant was collected, acidified to pH 2-4 with hydrochloric acid, and applied to a reverse phase octadecylsilane (C18) column (Supelco; Sigma), that had been previously washed with methanol and water. Captured HXA_3_ was rinsed with water and eluted in methanol, which was stored at − 80 °C until use. At time of use, HXA_3_ in methanol was dried under nitrogen gas and resuspended in HBSS to a final dilution of 1:4 of its starting volume.

### In vitro neutrophil transmigration assay

Primary neutrophils were purified from peripheral blood of healthy human volunteers as previously described [2]. Neutrophils were isolated using acid-citrate-dextrose anticoagulation and 2% gelatin sedimentation. Red blood cells were lysed in cold ammonium chloride buffer and neutrophils were washed with HBSS−/− (without Ca^2+^ or Mg^2+^ cations) and suspended to a final volume of 5 × 10^7^ neutrophils/mL in HBSS−/−.

T84 cells grown on the bottom-facing surface of collagen-coated 96-well Transwell® plates (5 μm pore, Costar), after 24 h incubation with metabolites as described above, were washed with warm HBSS and equilibrated in HBSS for 1 h at 37 °C and 5% CO_2_. HXA_3_ (prepared as described above) or HBSS alone was added to the bottom (“apical”) compartment of the Transwell® plate. HBSS was added to the top (“basolateral”) compartment, followed by 0.5 × 10^6^ primary neutrophils per well to the top compartment. Plates were incubated for 3 h at 37 °C and 5% CO_2_ to allow neutrophil migration to the bottom compartment, after which the wells were removed, and neutrophils in the bottom compartment were lysed with 0.5% Triton-X-100 for 30 min at 4 °C. Sodium citrate buffer (0.1 M, pH 4.2) was then added to the samples. A solution of 2,2′-azino-bis (3-ethylbenzothiazoline-6-sulphonic acid) (ABTS, Sigma) was prepared in sodium citrate buffer (0.1 M, pH 4.2), to which hydrogen peroxide was added at 1:1000 dilution. Equal volumes of neutrophil lysate and ABTS were mixed prior to measurement of absorbance at 405 nm. A standard curve of neutrophils was prepared alongside the migration samples and used to quantitate the number of neutrophils that migrated to the bottom compartment of the plate.

### LDH toxicity assay

Supernatants from T84 cells treated with metabolites were pulled after a 24-h incubation described above and stored at – 20 °C in storage buffer (200 mM Tris HCl pH 7.3, 10% Glycerol, 1% BSA) until further processing. To measure lactate dehydrogenase (LDH) release into the supernatants, LDH was measured by LDH-Glo Cytotoxicity Assay (Promega) following manufacturer instructions. As a positive control for toxicity, T84 cells incubated 24 h with 5% DMSO in growth media were included. This sample was not included in the statistical analysis. Samples of media only were included and LDH in the media was subtracted from all data points.

### TER measurement

Barrier integrity of T84 cells on 24-well Transwell® plates was measured by transepithelial resistance (TER) by using dual electrodes between the two compartments (World Precision Instruments). Fold change differences between measures of resistance (Ω) before and after metabolite incubation were calculated. As a positive control for toxicity and therefore drop in TER, T84 cells were incubated 24 h with 5% DMSO added to growth media. This sample was not included in the statistical analysis.

### Western blot

Protein lysates from colonic tissue (human or mouse) were generated using bead tubes containing Lysing Matrix D (MP Biomedical) and lysis buffer (20 mM Tris pH 7.4, 120 mM NaCl, 1 mM EDTA, 1% Triton-X-100, 0.5% sodium deoxycholate, 1× protease inhibitor cocktail [Roche]) in a benchtop homogenizer. Protein lysates from cell lines were generated by incubation in lysis buffer (above). Lysates were normalized for protein concentration using the DC Protein Assay (BioRad), separated by SDS-PAGE gels under reducing conditions, and transferred to nitrocellulose membranes. After 1 h incubation in PBS-based blocking buffer (LI-COR), blots were incubated overnight with primary antibodies anti-P-gp (C219, Millipore) at 1:500, anti-villin (Ab130751, Abcam) at 1:5000 or anti-GAPDH (MAB374, Millipore) at 1:40,000. After washing with PBST (PBS + 0.1% Tween), membranes were incubated 1 h in secondary antibody: IRDye® 800CW Goat anti-Mouse IgG (LI-COR) at 1:5000 (P-gp) or 1:40,000 (GAPDH) or IRDye® 680RD Goat anti-Rabbit IgG (LI-COR) at 1:10,000 (villin) . Membranes were scanned using an Odyssey Infrared Imaging System (LI-COR). Densitometry analysis was performed using Image Studio Lite Version 5.2. Densitometry values for P-gp were normalized to internal protein loading control GAPDH.

### Flow cytometry analysis of P-gp expression

Flow cytometry measurement of P-gp surface expression was performed as described previously [29]. Briefly, cells were lifted with 0.25% trypsin-EDTA (Gibco) for 15 min at 37 °C and suspended in cold 1× stain buffer (PBS, 3% FBS, 1 mM EDTA). Cells were incubated at 0.5 x 10^6^ cells per sample for 30 min in 100 μL stain buffer containing allophycocyanin (APC) anti-human P-gp UIC2 clone (BioLegend catalog no. 348607), or isotype control APC mouse IgG2a(k) (BioLegend catalog no. 400221). Cells were washed and resuspended in 4=, 6-diamidino-2-phenylindole (DAPI; Thermo) for live/dead differentiation. Cell suspensions were filtered through 40 μm nylon mesh prior to data collection on a MACSQuant10 flow cytometer (Miltenyi Biotec). Data were analyzed using the FlowJo software (TreeStar). The geometric mean of APC of the DAPI- population was computed for each sample and normalized to an untreated medium control sample, as well as a “colyte” solution (60 g/L PEG3350, 1.46 g/L sodium chloride, 0.74 g/L potassium chloride, 1.68 g/L sodium bicarbonate, 5.68 g/L sodium sulfate) as a representative bowel prep vehicle control.

### Human sample collection

Fecal samples, mucosal brushings, and colonic biopsy specimens were collected from the ascending colon during routine colonoscopy upon informed consent using a standard institutional approved IRB protocol during a planned clinical visit. Fecal samples were flash-frozen upon voiding and stored at – 80 °C. Mucosal brushings were processed as described below. Colonic biopsy specimens were flash-frozen upon collection and stored at − 80 °C.

### Lipid analysis of human mucosal brushings

Cytology brushes from human mucosal brushing collection were cut into glass tubes containing 4 mL PBS with 10 mM EDTA and kept on ice until further analysis. After vigorous shaking to remove mucosa from brush, then removal of the brush, 4 μg AEA-d8 (Cayman Chemical) as an internal standard was added to each sample, followed by 12 mL of a 2:1 chloroform: methanol solution (OmniSolv, Sigma). Tubes were shaken for 30 s, then samples were stored at 4 °C standing overnight to allow separation. The organic layer was then removed to a glass vial and dried down under a stream of nitrogen gas.

Lipid fractions were stored at − 80 °C until LC-MS analysis. Samples were resuspended in 1 mL 50 % acetonitrile (ACN) and 10 μL injected. Reverse-phase separation was performed on an Ultimate 3000 UPLC (Thermo Fisher Scientific), using an Acquity BEH C18 column (2.1 × 50 mm, 1.7 μm, Waters). The mobile phase consisted of A: ACN/water (30/70, *v/v,*) (VWR, HiPerSolv) and B: ACN (100) (VWR). Gradient elution was carried out with 0% mobile phase B until 1 min, followed by a linear gradient to 100% B at 6 min. 100% B was held to 10 min, returning to 0% B with a total run time of 14 min. The flow rate was 0.4 mL/min, with the column temperature kept at 50 ^o^C. All samples were kept at 4 ^o^C throughout analysis.

Mass spectrometry was performed using a MaXis HD QTOF-MS (Bruker Daltonik) operated in positive mode with the following parameters: capillary voltage 4500 V, nebulising gas 4 bar, drying gas 12 L/min at 220 ^o^C. The TOF scan range was 75–750 mass-to-charge ratio. The MS instrument was calibrated using a range of sodium formate clusters (prepared in-house) introduced by switching valve injection during the first minute of each chromatographic run. The mass calibration solution consisted of 3 parts of 1 M NaOH (VWR) to 97 parts of 50/50 water/isopropanol, *v/v* (VWR) with 0.2% formic acid (Fluka).

Data processing was carried out using Compass DataAnalysis 4.3 and Compass QuantAnalysis 4.3 (Bruker Daltonik). Lipids were identified by comparison of accurate mass (± 0.005 Da) and retention time (± 0.2 min) with commercial standards: 11(Z),14(Z)-Eicosadienoic Acid Ethanolamide (CAS no. 162758-92-1), Anandamide (AEA, CAS no. 94421-68-8), Oleoyl Ethanolamide (OEA, CAS no. 111-58-0), and Palmitoyl Ethanolamide (PEA, CAS no. 544-31-0) (Cayman Chemicals). Relative quantitation of lipids against internal standard (AEA-d8) was undertaken for [M+H]^+^ ions.

### Calprotectin ELISA of human fecal samples

Fifty to 100 mg of frozen human fecal samples stored at − 80 °C were weighed into sterile 5 mL polypropylene tubes. Extraction and measurement of calprotectin levels were performed using the fCAL ELISA kit (Bühlmann Diagnostics), following the manufacturer’s instructions. Dilutions of samples were modified accordingly to accurately quantitate within the range of the included standard curve.

### Fecal supernatant preparation

Human fecal supernatants were prepared as previously described [29]. Fecal samples stored at − 80 °C were weighed and resuspended in serum-free growth medium to 0.25 g/mL (wt/vol). Samples were homogenized, followed by centrifugation at 10,000×*g* for 15 min. The supernatant was sterile-filtered through a 0.22-μm polyethersulfone (PES) filter and diluted 10-fold in serum-free T84 growth medium before adding to the apical surface of T84 monolayers grown on transwell plates. Cells were incubated with fecal supernatants for 12 h at 37 °C and 5% CO_2_ prior to preparation for flow cytometry analysis of P-gp expression, as described above.

### Statistical analyses

Statistical tests as indicated in the figure legends were carried out using GraphPad Prism or R. All results are shown as box plots or column plots indicating mean ± standard deviation.

## Results

**Fig. 1:**
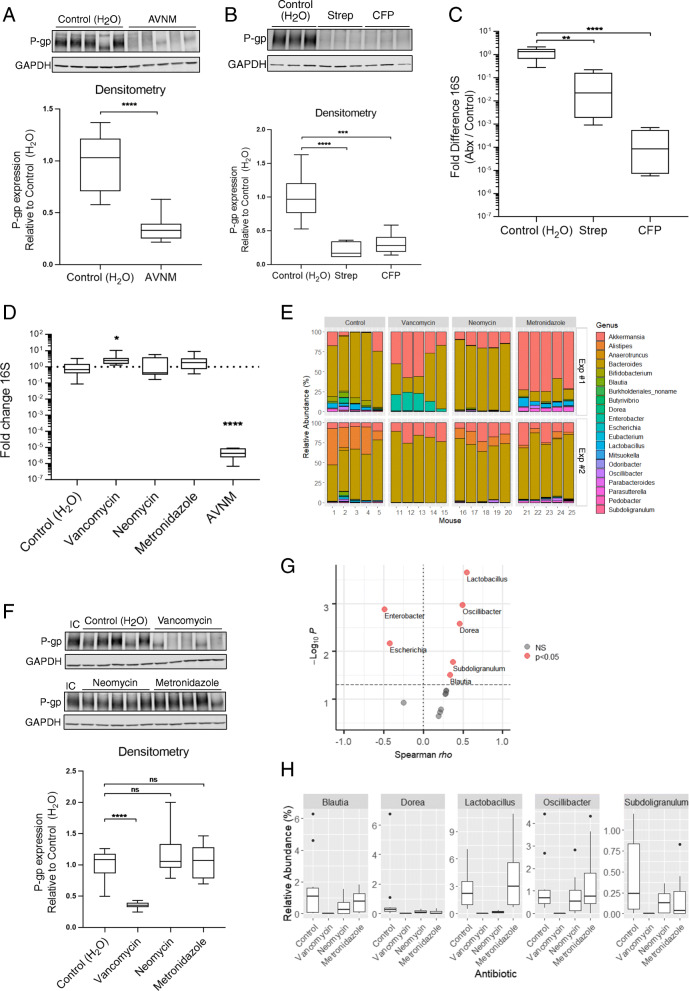
Vancomycin-sensitive gut microbiota is required for colonic P-gp expression. **A**, **D** WT SPF mice were treated with AVNM for 10 days. Representative western blot of P-gp protein expression in colonic tissue is shown, each lane representing a replicate mouse within each group. *N* = 10 mice per group; ****p < 0.0001, unpaired *t* test. **B**, **C** WT SPF mice were treated with streptomycin (Strep) or cefoperazone (CFP) for 7 days. **B** P-gp protein expression was measured as in **A***.* Representative western blot is shown, each lane representing a replicate mouse within each group. *N* = 6 mice per group; ***p = 0.0003, ****p < 0.0001, one-way ANOVA with Dunnett’s multiple comparisons test. **C** Fold difference changes in relative amount of 16S DNA in feces collected from mice on the last day of antibiotic treatment are shown. Data are pooled from two independent experiments, *N* = 6 per group ****p < 0.0001, **p = 0.0028 one-way ANOVA with Dunnett’s multiple comparisons test*,* statistical test for both based on ΔΔC_t_ values. **D** Fold difference in relative amount of 16S DNA in feces collected from mice on day 10 of antibiotic delivery (**A**, **E–H**), relative to control. Data are pooled from two independent experiments, *N* = 10 mice per group. *p = 0.0178, ****p < 0.0001, one-way ANOVA with Dunnett’s multiple comparisons test, based on ΔΔ C_t_ values. **D–H** WT SPF mice were treated with vancomycin, neomycin, metronidazole, or AVNM for 10 days. **E** Relative abundance of bacterial genera in mouse fecal microbiota. Representative plot from each of two independent experiments is shown. *N* = 5 per group, per experiment. **F** P-gp protein expression was measured as in (**A**, **B**)*.* An internal control (“IC”) lysate was included across multiple blots for normalization. Representative western blot is shown, each lane representing a replicate mouse within each group. *N* = 10 mice per group; ****p < 0.0001, ns *p* > 0.05, one-way ANOVA with Dunnett’s multiple comparisons test. **A**, **B**, **F** Densitometry data describe samples pooled from two independent experiments. **G** Bacterial genera positively or negatively correlated with colonic P-gp expression by Spearman correlation test of sequencing data from experiments performed in Fig. 1, significance indicated by *p* < 0.05. **H** Relative abundance of bacterial genera positively correlated with colonic P-gp expression shown in **G**

We sought to determine the role of the intestinal microbiota in driving colonic P-gp expression and function in gastrointestinal homeostasis. We began by clearing the microbiota in conventional wild-type mice with broad-spectrum antibiotic treatment. Specifically, treatment with the antibiotic cocktail AVNM (ampicillin, vancomycin, neomycin, and metronidazole) resulted in a sustained reduction of both colonic P-gp expression and microbial load through day 10 of antibiotic treatment (Fig. 1A, D, Figure S1A–D). This was not an artifact of the AVNM cocktail given as each of the broad-spectrum antibiotics streptomycin or cefoperazone also reduced colonic P-gp expression and intestinal microbial load (Fig. 1B, C). To define the microbial community member(s) responsible for maintenance of P-gp expression, we utilized the specificity of each AVNM cocktail component to selectively deplete subsets of the mouse intestinal microbiota. As expected, metagenomic analyses revealed that treatment of conventional wild-type mice with either vancomycin, neomycin, or metronidazole for 10 days demonstrated perturbations in their microbiota (Fig. 1E), without affecting total bacterial load (Fig. 1D). Ampicillin treatment of mice had broad-spectrum activity that reduced bacterial load (Figure S2A) and, thus, was not pursued further.

Colonic tissue from mice treated with vancomycin, but not from those treated with neomycin or metronidazole, showed significant reduction in P-gp protein expression (Fig. 1F). We identified five genera that positively correlate, and two genera that negatively correlate, with colonic P-gp expression in these mice, suggesting they may influence P-gp protein expression (Fig. 1G, H, Table S1). Since our results with broad spectrum antibiotics (Fig. 1A–D) indicate presence of bacteria that positively regulate P-gp expression, we pursued genera within the *Bacilli* and *Clostridia* classes that showed a positive correlation with P-gp expression (Fig. 1G, H, Table S1). Consistent with these observations, culture supernatants from *Lactobacillus* species are sufficient to induce P-gp expression in vitro [30]. To control for possible morphological changes to the intestinal epithelium with antibiotic treatment (e.g., cell shedding) [27], we found expression of the epithelial marker villin did not change in the colonic tissue of these mice (Figure S2B), indicating a specific loss of P-gp expression in the epithelium. Additionally, antibiotics did not directly affect P-gp expression in vitro in T84 intestinal epithelial cells (Figure S2C).

**Fig. 2:**
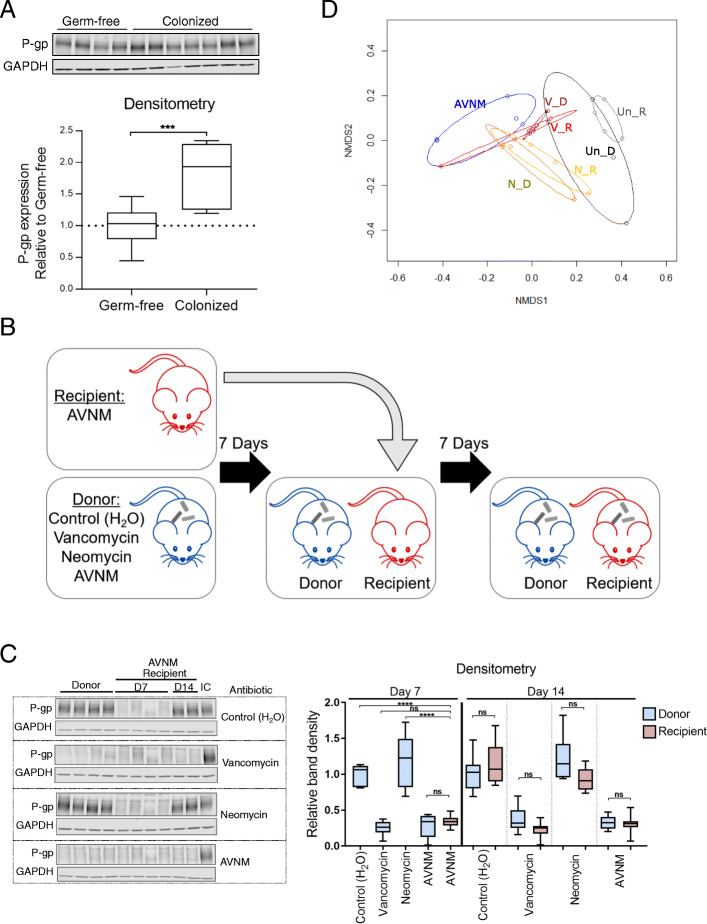
Colonization of mice with gram-positive microbiota is sufficient to induce P-gp expression. **A** WT germ-free mice were colonized with cecal contents from a WT donor mouse. After 14 days, P-gp protein expression was measured in colonic tissue by western blot. *N* = 7–10 mice per group; ***p = 0.0005, unpaired *t* test. **B** Schematic of reconstitution by coprophagy for experiments shown in **C**, **D**. WT “donor” mice were treated with antibiotic for 7 days. AVNM-treated WT “recipient” mice were then cohoused with “donor” mice from each of the other groups for 7 days. **C** P-gp protein expression was measured as in **A**. Representative western blot is shown, each lane representing a replicate mouse within each group. An internal control (“IC”) lysate was included across multiple blots for normalization. *N* = 6–8 mice per group; ****p < 0.0001 by one-way ANOVA with Tukey’s multiple comparisons test. **A**, **C** Densitometry data describe samples pooled from two independent experiments. **D** Bray-Curtis based nonmetric multidimensional scaling (NMDS) ordination of fecal microbiota from mice in **B**, **C**. Each dot represents an individual mouse from one representative experiment, with treatment groups denoted by color: Un_R untreated recipient, Un_D untreated donor, V_R vancomycin recipient, V_D vancomycin donor, N_R neomycin recipient, N_D neomycin donor, and AVNM

Since these data indicate that the intestinal microbiota is required for governing the expression of P-gp in the colon, we next examined whether the intestinal microbiota is also sufficient to induce P-gp expression. As expected, colonization of germ-free mice with cecal microbiota from wild-type conventionally raised mice induced colonic P-gp protein expression (Fig. 2A). To stratify the microbiota community sufficient for inducing P-gp protein expression, we performed microbiota reconstitution assays using antibiotic treated donor mice. Recipient mice were first cleared of the microbiota using the AVNM cocktail, then cohoused with donor mice treated with single antibiotics (Fig. 2B). Coprophagy by the recipient served as a mechanism for microbiome reconstitution, modeled after previous studies [31]. Remarkably, AVNM-treated recipient mice co-housed with either untreated or neomycin-treated donors, regained intestinal P-gp expression, while AVNM-treated recipient mice co-housed with either vancomycin- or AVNM-treated donors remained devoid of P-gp expression (Fig. 2C). We confirmed recipient recolonization of bacterial subpopulations that aligned with that of the donor (Fig. 2D), consistent with the hypothesis that the vancomycin-sensitive bacterial community is necessary and sufficient to induce intestinal P-gp expression in vivo.

**Fig. 3:**
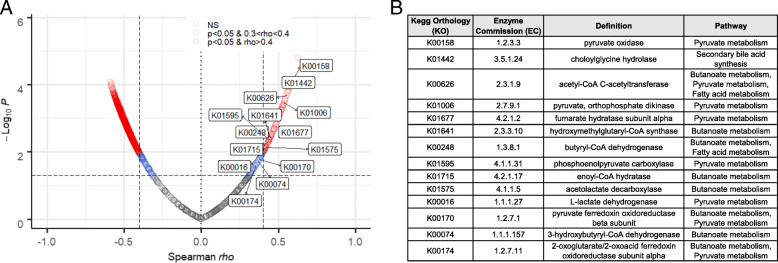
Short-chain fatty acid- and secondary bile acid-producing bacteria positively correlate with colonic P-gp expression. **A** Volcano plot showing the *rho* coefficient and corresponding *p* value of Spearman’s correlation test of relative abundance of microbial genes from whole genome sequencing data (regrouped to KO groups) relative to expression of P-gp by western blot densitometry in each mouse. Significance set at *p* < 0.05. KOs involved in butyrate and secondary bile acid metabolism pathways are labeled. **B** Positively correlated KOs identified in **A** with corresponding enzyme commission (EC) numbers, definition, and involved pathways being indicated

**Fig. 4:**
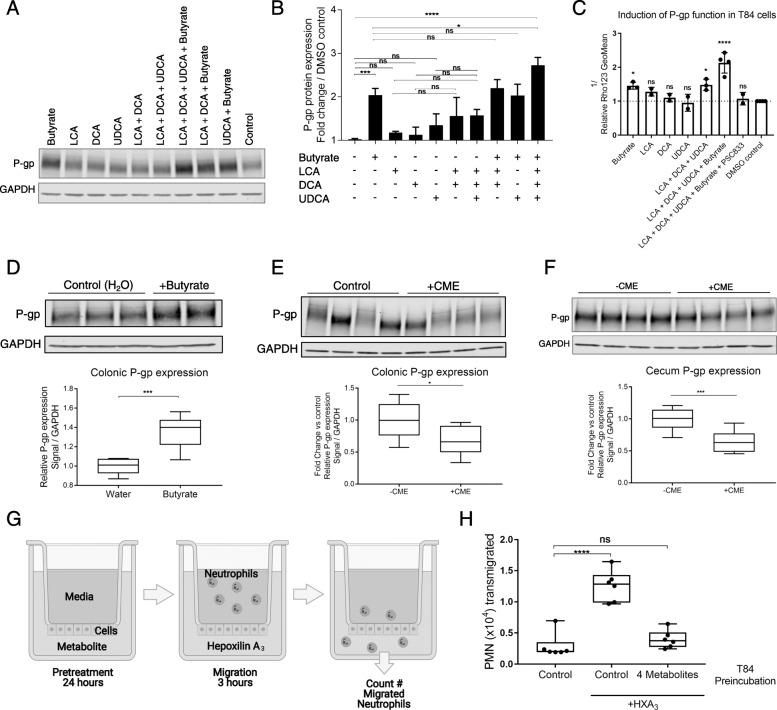
Butyrate and secondary bile acids induce P-gp to limit neutrophil transmigration across the epithelium. **A–C** T84 cells incubated with 5 mM butyrate, 50 μM LCA, 50 μM DCA, and/or 50 μM UDCA for 24 h. **A** Representative western blot showing P-gp expression in lysates. **B** Densitometry data describe samples pooled from at least two independent experiments; *p = 0.0199, ***p = 0.0002, ****p < 0.0001, one-way ANOVA with Tukey’s multiple comparisons test. **C** T84 cells were treated as in **A**, **B**, prior to measurement of Rho123 retention. Data reflect inverse of geometric mean of Rho123 fluorescence intensity. Data describe samples pooled from at least two independent experiments; *p < 0.05, ****p < 0.0001, one-way ANOVA with Dunnett’s multiple comparisons test of each sample compared to DMSO control. **D** WT germ-free mice were delivered butyrate orally for 14 days. P-gp protein expression was measured in colonic tissue by western blot. Representative western blot is shown, each lane representing a replicate mouse within each group. Densitometry data describe samples pooled from two independent experiments; *N* = 6–7 mice per group; ***p = 0.0004, unpaired *t* test. **E**, **F** WT SPF mice were delivered cholestyramine (CME) in mouse chow for 14 days. P-gp protein expression was measured by western blot in colon tissue (**E**) or cecum tissue (**F**)**. E**, **F** Representative western blot is shown, each lane representing a replicate mouse within each group. Densitometry shown relative to control group without cholestyramine. Data describe samples pooled from two independent experiments. *N* = 8 mice per group. **E** *p = 0.0237, unpaired *t* test. **F** ***p = 0.0009, unpaired *t* test. **G** Schematic of neutrophil migration experiment (BioRender). T84 cells seeded on the bottom-facing surface of Transwell® plates were preincubated with metabolites for 24 h. Primary neutrophils were added to the top (basolateral) compartment, purified HXA_3_ was added to the bottom (apical) compartment, and the number of primary neutrophils that migrated across the T84 cell monolayer were quantitated. **H** T84 cells seeded as in **G** were incubated with butyrate, LCA, DCA, and/or UDCA as in **A–C**. Migration of primary neutrophils across this cell monolayer to HXA_3_ in the apical compartment was quantified. Data shown indicate individual replicate wells and are representative of at least three independent experiments; ****p < 0.0001, ns *p* > 0.05, one-way ANOVA with Tukey’s multiple comparisons test

Metagenomic analysis comparing vancomycin-treated and untreated microbiota revealed Kegg Orthology (KO) groups involved in short-chain fatty acid and secondary bile acid production among those highly correlated with colonic P-gp expression (Fig. 3A, B, Data S1). These classes of microbial-derived metabolites are linked to intestinal barrier integrity, immune response modulation, and metabolism [32–36]. Butyrate, one of the most abundant short-chain fatty acids in the intestine, has been shown to promote P-gp expression in cancer studies [37, 38]. We demonstrated that physiological levels of butyrate induce both P-gp expression and function in vitro, as determined by western blot and efflux of the P-gp substrate Rhodamine123, respectively (Fig. 4A–C). In contrast, acetate and propionate did not significantly affect P-gp expression (Figure S3A,B). Moreover, butyrate alone was sufficient to induce P-gp expression in germ-free mice (Fig. 4D).

Secondary bile acids are also linked to intestinal health via anti-inflammatory activities including promotion of regulatory T cells and suppression of dextran sodium sulfate (DSS)-induced colitis [32, 33]. Three of the most abundant, lithocholic acid (LCA), deoxycholic acid (DCA) and ursodeoxycholic acid (UDCA), had an additive effect for inducing P-gp expression and function in vitro (Fig. 4A–C). Treatment of mice with cholestyramine to sequester bile acids [26] altered secondary bile acid absorption in the colon, particularly UDCA (Figure S4), and significantly reduced P-gp expression in both the cecum and colon (Fig. 4E, F), suggesting a requirement for UDCA for full expression of P-gp.

Although our in vivo studies suggest either butyrate or secondary bile acids can independently induce P-gp expression, each study demonstrated a relatively modest effect (Fig. 4D–F). In support of the hypothesis that intestinal homeostasis depends on functional cooperativity of a larger complex microbial community and its metabolites, in vitro testing revealed a striking potentiation of the induction of P-gp expression in T84 cells when exposed to a combination of butyrate and all three bile acids LCA, DCA, and UDCA (Fig. 4A, B). By contrast, a combination of butyrate with LCA and DCA, or with UDCA alone, was not sufficient to observe this effect, indicating a requirement for all three bile acids (Fig. 4A, B). In particular, the observed requirement for UDCA for full induction of P-gp expression mimics what we observed in vivo with bile acid sequestration (Fig. 4E, F). Furthermore, we assessed P-gp function in T84 cells by measuring Rhodamine123 efflux, which was maximal after incubation with the four metabolites (butyrate, LCA, DCA, UDCA) and blocked by the P-gp selective inhibitor PSC833 (Fig. 4C). Remarkably, induction of P-gp activity by the four-metabolite combination also suppressed neutrophil transepithelial migration driven by the potent chemoattractant HXA_3_ (Fig. 4G, H), an outcome that was independent of cellular toxicity or changes to transepithelial resistance (TER) (Figure S3C, D).

**Fig. 5:**
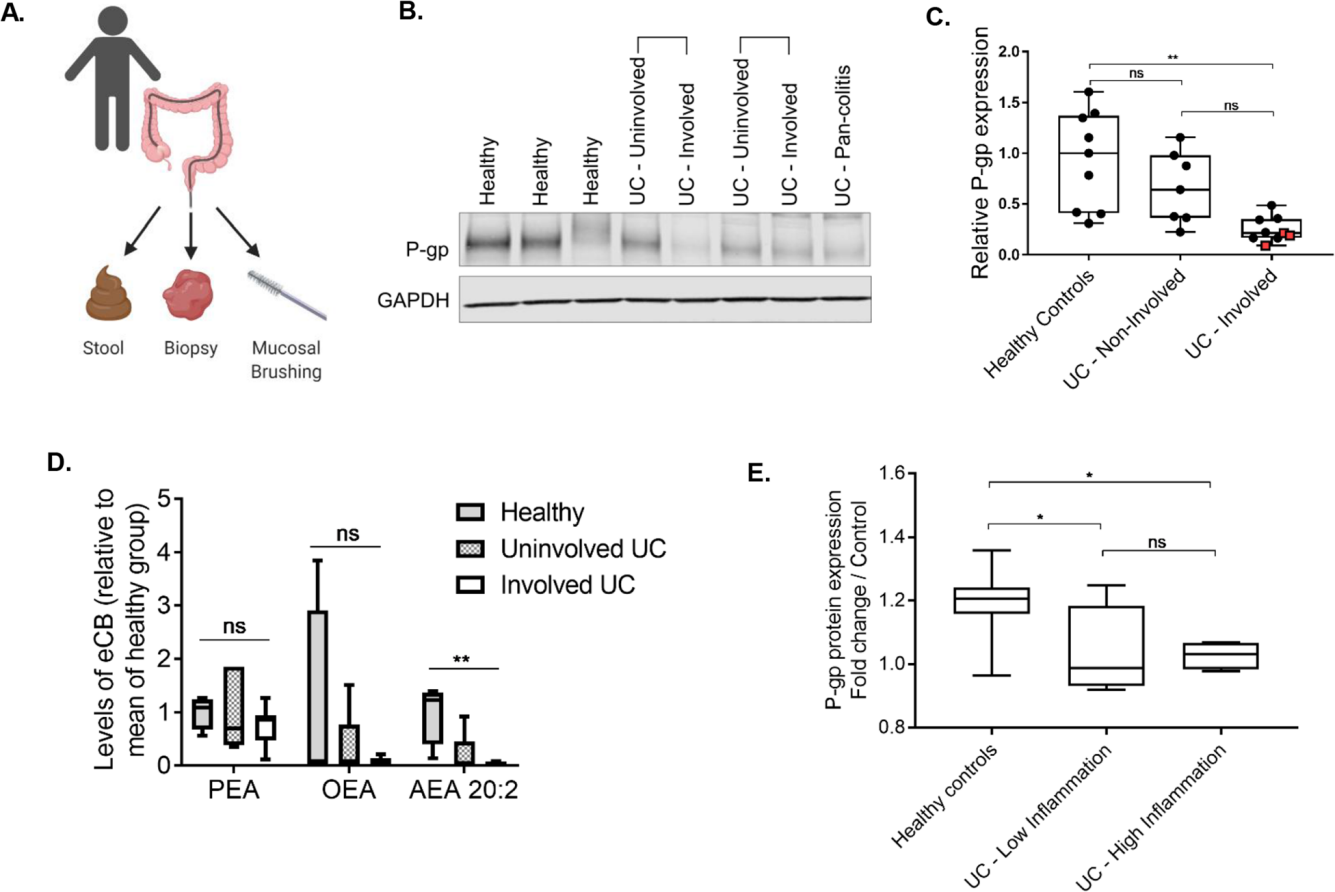
Human UC intestinal contents are unable to maintain P-gp expression. **A** Diagram of human sample collection (BioRender). **B** Representative western blot of P-gp expression in human patient colon biopsies, showing healthy control versus UC patient, and uninvolved versus involved tissue, with samples from same UC patients indicated with brackets. **C** Densitometry data of **B***, N* = 7–9 patients per group, red squares indicate 3 pan-colitis patients; **p = 0.0012, one-way ANOVA with Tukey’s multiple comparisons test. **D** Palmitoyl ethanolamide (PEA), oleoyl ethanolamide (OEA), and anandamide (AEA) 20:2 measured in mucosal brushings of human patients. *N* = 4 healthy controls, *N* = 7–8 UC patients. **p = 0.0013, one-way ANOVA with Tukey’s multiple comparisons test. **E** Functional P-gp expression induced by human patient fecal samples in T84 cells in vitro. Data are normalized to representative vehicle control bowel prep solution; *N* = 5–11 patients per group; *p < 0.05, one-way ANOVA with Tukey’s multiple comparisons

These findings were also corroborated in clinical samples from a cohort of UC patients suffering from chronic colonic inflammation. Colonic tissue biopsies, mucosal brushings, and fecal samples were collected during routine colonoscopy procedures (Fig. 5A, Table S2). From patients with active disease, we collected biopsies and mucosal brushings from both involved (inflamed) or uninvolved (non-inflamed) regions of the colon. We observed that P-gp expression was significantly diminished in UC patients compared to healthy controls, and moreover, was decreased in involved regions versus uninvolved regions of the same patient (Fig. 5B, C), consistent with previous reports [9, 39]. With the exception of four patients with severe/pan-colitis (> 1000 μg/g calprotectin, Table S2) and presumed damage to the epithelium, P-gp expression did not significantly correlate with villin expression, again supporting specific loss of P-gp expression (Figure S5A, B). Lipidomic analysis of mucosal brushings confirmed that anandamide (AEA), the predominant eCB secreted by P-gp that suppresses neutrophil infiltration into the intestinal lumen [2], was decreased in involved regions versus uninvolved regions of the same patient (Fig. 5D).

Thus far, our data indicate P-gp and its eCB substrates are reduced in the epithelium of UC patients, suggesting loss of a positive regulator of P-gp. We next sought to determine whether luminal components actively drive P-gp expression. Soluble stool fractions from healthy controls, but not UC patients, significantly induced functional P-gp expression in T84 intestinal epithelial cells (Fig. 5E). Notably, there was no significant difference in P-gp induction by soluble fecal samples from patients with high versus low inflammation, as binned by fecal calprotectin levels (Fig. 5E, Table S2), indicating the soluble fraction of fecal samples from UC patients, regardless of the current state of inflammation, have reduced potential to induce colonic P-gp expression. Consistent with our observations, a recent study also found enrichment of members of the *Clostridia* class (namely species of the *Lachnospiraceae* family) in healthy controls compared to UC patients [40]. The functional potential of these family members also aligns with that of the mouse microbiome identified in these studies (Figs. 1E, G, H, 3, Table S1). Altogether, these data strengthen our findings that microbial-derived short-chain fatty acids and secondary bile acids play a fundamental role in induction of P-gp expression and function.

## Discussion

P-gp has been well-studied as a transporter at the intestinal epithelial surface that functions to efflux toxins and xenobiotic compounds, including chemotherapeutics [3]. We have recently identified a new role for P-gp in exporting NAE-class endocannabinoid molecules that suppress neutrophil transmigration across the intestinal epithelium [2]. The importance of functional P-gp expression at the epithelial surface is underscored by the association of dysfunctional P-gp with increased susceptibility to inflammatory bowel diseases including UC [4, 5], consistent with the observed development of spontaneous colitis in *mdr1a*−*/*− mice [6–8].

Here, we identify a subpopulation of the intestinal microbiome community, including genera within the *Clostridia* and *Bacilli* classes, that is required and sufficient to induce colonic P-gp expression, demonstrated in the microbiota of mice through studies of antibiotic perturbation and reconstitution after antibiotic clearance. This bacterial community has functional potential to produce short-chain fatty acids such as butyrate from dietary fiber and to convert primary bile acids to secondary bile acids. In a cohort of UC patients, we first confirmed previous studies showing reduction of P-gp expression in inflamed tissue [9, 10], but also showed that this expression is mirrored by a reduction in the mucosa of one of the primary NAE-type endocannabinoids secreted by P-gp, AEA. Furthermore, we found that the luminal content contained in stool samples from healthy controls, but not UC patients, had functional capacity to induce P-gp expression ex vivo in the T84 intestinal epithelial cell line, pointing to a soluble factor deriving from a healthy microbiota.

Butyrate is one of the three most abundant short-chain fatty acids, along with acetate and propionate, and is present at millimolar concentrations in the healthy intestine. Importantly, butyrate also serves as a major energy source for the intestinal epithelium, promotes colonic regulatory T cell differentiation, and enhances barrier function [34–36, 41]. It is currently unclear whether butyrate and P-gp function through entwined versus independent pathways to promoter barrier integrity [7, 8]. Moreover, P-gp secreted endocannabinoids may signal through cannabinoid receptor 1 (CB1) in the epithelium to promote epithelial barrier function, as has been reported for similar classes of endocannabinoids [42, 43]. Additionally, short-chain fatty acids contribute to resident microbiome resistance to infection by pathogens including *Salmonella typhimurium* [44, 45]. Though reports suggest this may involve direct downregulation of *S. typhimurium* pathogenicity genes, this may also involve P-gp, as overexpression of P-gp in intestinal epithelial cells in vitro increases resistance to *S. typhimurium* infection [46].

**Fig. 6:**
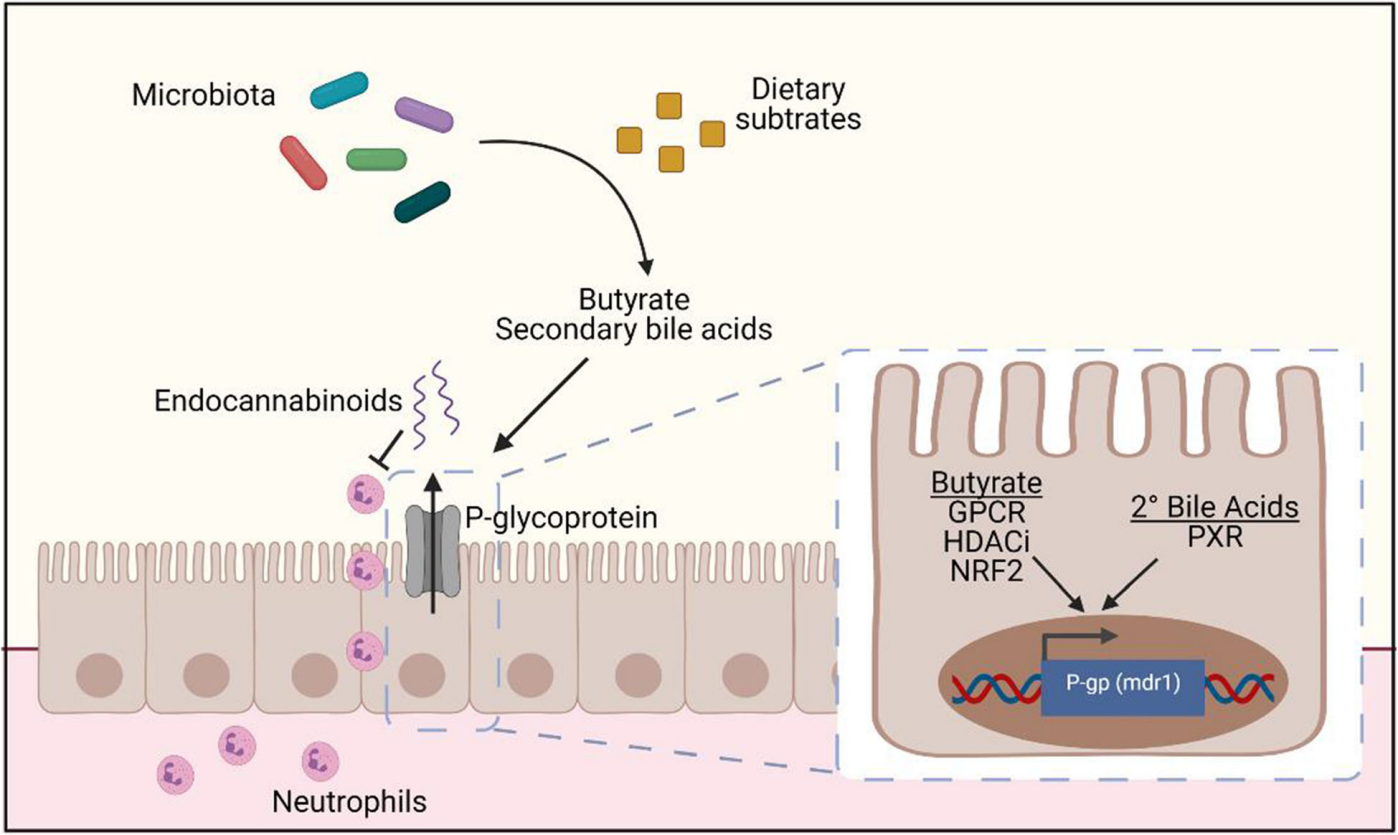
A working model of microbiome-driven P-gp expression. We have identified a bacterial population containing Bacilli and Clostridia classes that is sufficient to induce P-gp expression in the colon. Butyrate and secondary bile acids produced by these bacteria from dietary substrates together potentiate the induction of functional P-gp expression on the epithelium which is capable of blocking neutrophil migration through efflux of its endogenous substrates, endocannabinoids [2]. Our observations suggest converging intracellular pathways to amplify P-gp expression; we propose these pathways include one or more of the following: GPCR activation, HDAC inhibition, NRF2 activation, PXR activation. Schematic made with BioRender

Secondary bile acids including LCA, DCA, and UDCA are produced from bacterial conversion of primary bile acids by biosynthetic enzymes including the *bai* operon [47, 48]. These secondary bile acids have been shown to contribute to intestinal homeostasis via suppression of IL-17-producing T helper cells (T_H_17 cells) in the colon [33] and reduce inflammation in chemical-induced mouse models of colitis [16, 32]. Here, we show that the three bile acids LCA, DCA, and UDCA are capable of inducing P-gp expression and function in vitro. Notably, at the doses tested, these bile acids were individually insufficient for this effect. While butyrate has been shown to induce P-gp transcription in the context of various cancer models [37, 38], we observed that butyrate and these three secondary bile acids act in concert to promote maximal expression of functional P-gp. Though the precise mechanism remains unclear, prior studies have suggested at least one possible pathway involves butyrate inhibition of histone deacetylases (HDACi) to alter transcription [49, 50]. Additionally, butyrate may activate G-protein coupled receptors (GPCR) on the cell surface [11, 41, 51–53], or signal through transcription factors including nuclear factor erythroid 2-related factor 2 (Nrf2) [54]. Secondary bile acids can act as agonists of the nuclear receptor pregnane X receptor (PXR) [55]. Activation of Nrf2 and PXR by agonists have been shown to increase P-gp transcription [55–58]. Moving forward, we propose a model by which microbiota-derived butyrate and secondary bile acids activate receptors and signaling pathways that converge to induce P-gp transcription, thereby amplifying P-gp expression when both classes of microbiota-derived metabolites are present (Fig. 6). In addition, P-gp undergoes extensive post-translational modifications that contribute to its stabilization and activity [59]; whether these modifications occur downstream of butyrate and secondary bile acid signaling requires further investigation.

## Conclusion

This study provides a mechanistic link between metabolites produced by the healthy intestinal microbiome and suppression of neutrophil migration in UC, via induction of functional P-gp expression. For the first time, we show an example of two classes of metabolites, short-chain fatty acids, and secondary bile acids, acting in concert to regulate epithelial function. This reflects the importance of a specific collection of functional microbiome elements that cooperate to produce an output necessary for a homeostatic condition, distinct from requiring presence of specific taxa to effect such an outcome. This is further supported by recent findings that bacterial taxa across different phyla can contribute genes of a common function [60]. Therefore, our data suggest that nourishing the larger microbial community is critical and may be attained by processes known to impact the microbiome and its metabolite production [61, 62]. While dietary fiber can increase short-chain fatty acid production in the colon [63], specific dietary substrates required to support a microbiome supportive of colonic P-gp expression sufficient to limit overactive immune responses and maintain homeostasis are yet to be identified.

## Supplementary Information

**Additional file 1: Figure S1.** Intestinal bacterial load and P-gp expression are both reduced within 5 days of antibiotic treatment. **A** WT SPF mice were treated with AVNM cocktail for 5 days. One group of mice were euthanized on each day. A representative blot of P-gp protein expression in colonic tissue is shown, each lane representing a replicate mouse within each group. An “internal control” (IC) lysate was included across multiple blots for data comparison. **B** Densitometry data are shown for experiments performed in (A)*,* pooled from two independent experiments, *N* = 11 mice per group. ***p = 0.0001, ****p < 0.0001, one-way ANOVA with Dunnett’s multiple comparisons test. **C** Fold differences in relative amount of 16S DNA in feces collected from mice on Days 0-5 of AVNM delivery are shown for data pooled from two independent experiments, *N* = 11 mice per group. ****p < 0.0001, one-way ANOVA with Dunnett’s multiple comparisons test, based on ΔΔ C_t_ values. **D** Fold change differences in relative 16S DNA data are shown from **C** overlaid with relative P-gp protein expression from densitometry **B***,* with data representing the mean. **Figure S2.** Individual antibiotic treatment does not affect P-gp expression directly or through epithelial loss. **A** WT SPF mice were treated with ampicillin for 10 days, as in Fig. 1. Fold difference in relative amount of 16S DNA in feces collected from mice on Day 10 of antibiotic delivery, relative to control. Data from two independent experiments are shown individually with ampicillin treatment compared to control. *N* = 5 mice per group per experiment; ****p < 0.0001, two-way ANOVA with Tukey’s multiple comparisons test*.* **B** Representative western blot showing Villin expression in colonic tissue from mice treated with antibiotics as in **A** and Fig. 1, each lane representing a replicate mouse within each group. Densitometry calculated relative to control group; *N* = 3 per group, ns *p* > 0.05, one-way ANOVA. **C** Representative western blot showing P-gp expression in T84 lysates after incubation with antibiotic for 24 hrs. Densitometry calculated relative to media control samples. Data are pooled from two independent experiments; ns *p* > 0.05, one-way ANOVA. **Figure S3.** Short-chain fatty acids and secondary bile acids induce P-gp expression without affecting toxicity and TER. **A** Representative western blot of P-gp expression in T84 cells incubated with acetate, propionate, butyrate, or a combination of the three short-chain fatty acids (SCFAs) at a range of concentrations, all in physiologically relevant ratios. **B** Densitometry data for samples pooled from two independent experiments are compared to media control lysate; *p < 0.05, **p < 0.01, ***p < 0.001, one-way ANOVA with Tukey’s multiple comparisons test. **C** LDH release into supernatants from T84 cells incubated with butyrate and/or bile acids at the indicated concentrations for 24 hrs are shown. 5% DMSO was included as a positive control for toxicity. Data are samples pooled from two independent experiments; ns *p* > 0.05, one-way ANOVA. **D** Percent change in TER measured in transwell plates of T84 cells before and after 24hr incubation with butyrate and/or bile acids at the indicated concentrations. 5% DMSO was included as a control for toxicity and/or TER reduction. Data are shown for samples pooled from two independent experiments; ns *p* > 0.05, *p < 0.05, one-way ANOVA with Tukey’s multiple comparisons test. **Figure S4.** Bile acid sequestration in the intestine. Bile acid content of intestinal tissue from colon or cecum after delivery of cholestyramine (CME) in the chow for 14 days. Data shown are pooled from two independent experiments; *N* = 7-8 per group; **p < 0.01, ***p < 0.0001 for unpaired *t*-test. **Figure S5.** P-gp expression is decreased specifically with increasing inflammation in UC patients. **A** Representative western blot of villin expression in human patient colon biopsies, as in Fig. 5. **B** Densitometry data for relative villin expression compared to that of P-gp, color coded by patient sample type – healthy control, UC uninvolved, UC involved. There appears to be a correlation between P-gp expression and villin expression (left), however significance is lost when pan-colitis patients with severe inflammation and presumed wholesale epithelial damage are removed (right). **Table S1.** Spearman correlation results of bacterial genera and P-gp. Shown are *p*-values and *rho* coefficients from Spearman correlation tests of each bacterial genus identified through whole genome sequencing aligned with relative colonic P-gp expression data generated from western blot densitometry. **Table S2.** Human patient metadata. Shown are sex, age, diagnosis, and fecal calprotectin levels for each human patient from which samples were tested for P-gp expression in biopsies (Fig. 5B,C), endocannabinoid content in mucosal brushings (Fig. 5D) or P-gp induction by fecal samples (Fig. 5E). Patients were binned by calprotectin levels determined by ELISA into three groups – high inflammation, low inflammation, and no inflammation.

**Additional file 2: Data S1.** Kegg orthology (KO) groups correlated with P-gp expression. Shown are *p*-values and *rho* coefficients from Spearman correlation test of each KO group identified through whole genome sequencing that are significantly (*p* < 0.05) correlated (positively or negatively) with P-gp expression data generated from western blot densitometry.

## Data Availability

Access to databases and associated software tools generated under the project will be available for educational, research, and non-profit purposes. Raw sequencing reads from the mouse microbiome metagenomic analyses are available in the NCBI Sequencing Read Archive (SRA) under BioProject PRJNA719426.
