# Supplementary material for: Gut microbiota regulation of P-glycoprotein in the intestinal epithelium in maintenance of homeostasis
Source: Microbiome. 2021 Sep 7;9:183. doi: 10.1186/s40168-021-01137-3 (PMC8425172; doi:10.1186/s40168-021-01137-3)
Supplement: Supplementary file 2 — Additional file 1: Figure S1. Intestinal bacterial load and P-gp expression are both reduced within 5 days of antibiotic treatment. A WT SPF mice were treated with AVNM cocktail for 5 days. One group of mice were euthanized on each day. A representative blot of P-gp protein expression in colonic tissue is shown, each lane representing a replicate mouse within each group. An “internal control” (IC) lysate was included across multiple blots for data comparison. B Densitometry data are shown for experiments performed in (A), pooled from two independent experiments, N = 11 mice per group. ***p = 0.0001, ****p < 0.0001, one-way ANOVA with Dunnett’s multiple comparisons test. C Fold differences in relative amount of 16S DNA in feces collected from mice on Days 0-5 of AVNM delivery are shown for data pooled from two independent experiments, N = 11 mice per group. ****p < 0.0001, one-way ANOVA with Dunnett’s multiple comparisons test, based on ΔΔ Ct values. D Fold change differences in relative 16S DNA data are shown from C overlaid with relative P-gp protein expression from densitometry B, with data representing the mean. Figure S2. Individual antibiotic treatment does not affect P-gp expression directly or through epithelial loss. A WT SPF mice were treated with ampicillin for 10 days, as in Fig. 1. Fold difference in relative amount of 16S DNA in feces collected from mice on Day 10 of antibiotic delivery, relative to control. Data from two independent experiments are shown individually with ampicillin treatment compared to control. N = 5 mice per group per experiment; ****p < 0.0001, two-way ANOVA with Tukey’s multiple comparisons test. B Representative western blot showing Villin expression in colonic tissue from mice treated with antibiotics as in A and Fig. 1, each lane representing a replicate mouse within each group. Densitometry calculated relative to control group; N = 3 per group, ns p > 0.05, one-way ANOVA. C Representative western blot showing P-gp expression i [file 40168_2021_1137_MOESM2_ESM.pdf]

Supplementary Figure 1.

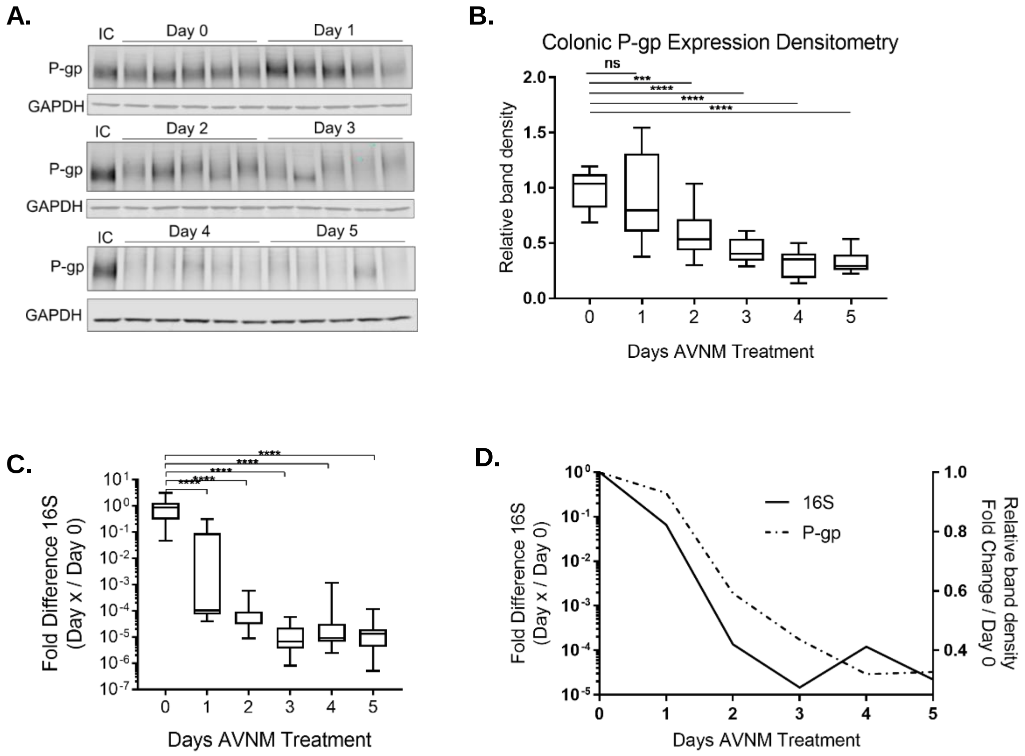

Supplementary Figure 2.

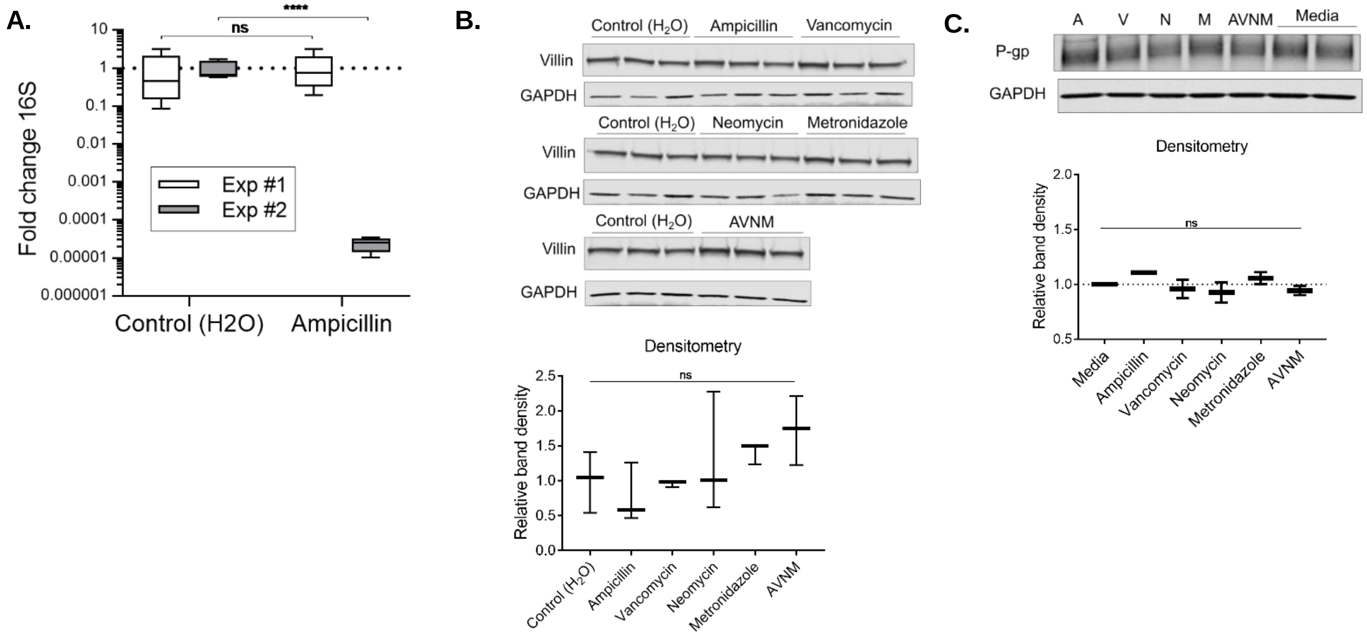

Supplementary Figure 3.

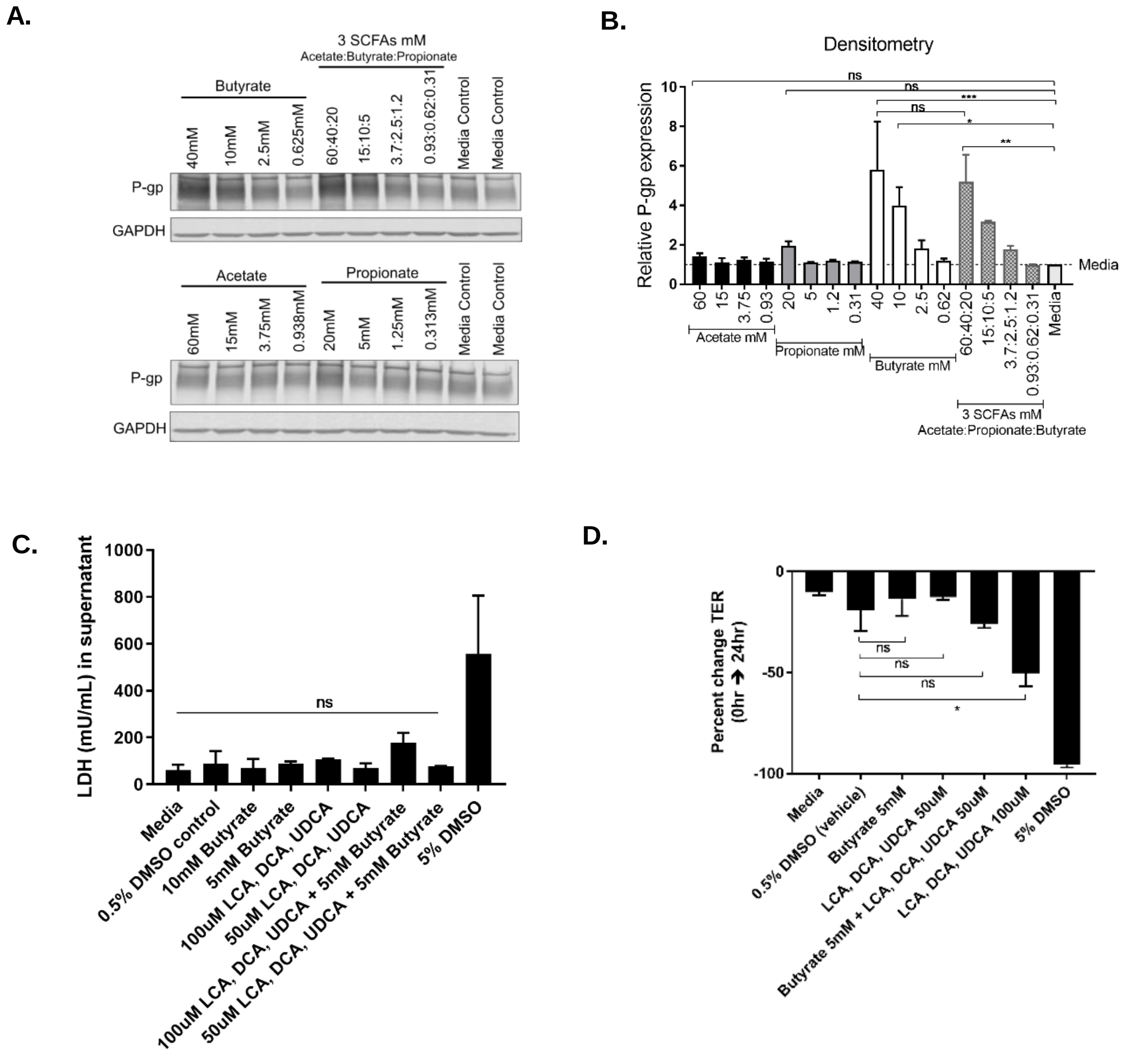

Supplementary Figure 4.

A.

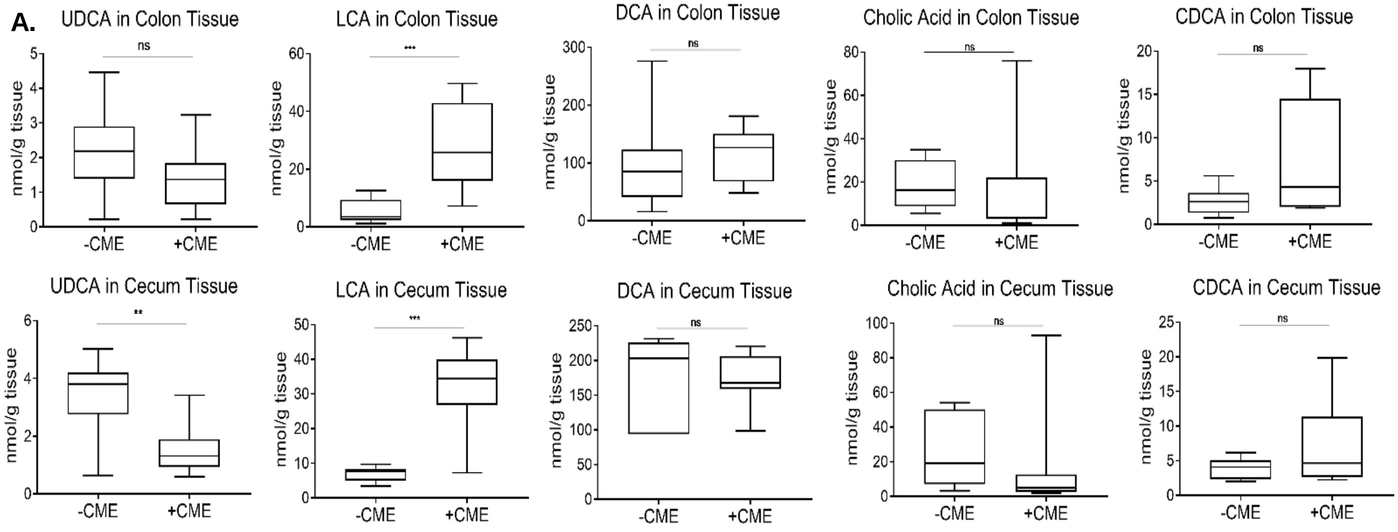

Supplementary Figure 5.

A.

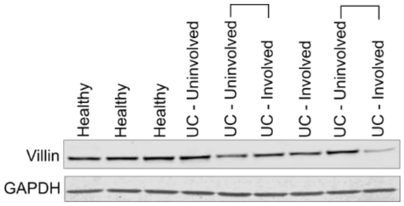

B.

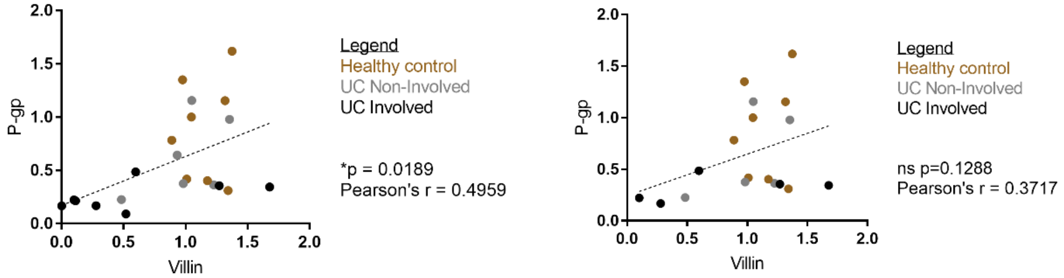

Supplementary Table 1

Phylum	Class	Order	Family	Genus	pvalues	rhovalues
Actinobacteria	Actinobacteria	Bifidobacteriales	Bifidobacteriaceae	Bifidobacterium	0.166884188	0.222865382
Bacteroidetes	Bacteroidia	Bacteroidales	Bacteroidaceae	Bacteroides	0.500314679	-0.109380863
Bacteroidetes	Bacteroidia	Bacteroidales	Porphyromonadaceae	Odoribacter	0.347564815	-0.152484311
Bacteroidetes	Bacteroidia	Bacteroidales	Porphyromonadaceae	Parabacteroides	0.558859343	-0.095232715
Bacteroidetes	Bacteroidia	Bacteroidales	Rikenellaceae	Alistipes	0.067362036	0.292147744
Bacteroidetes	Sphingobacteriia	Sphingobacteriales	Sphingobacteriaceae	Pedobacter	0.120764092	-0.249339167
Firmicutes	Bacilli	Lactobacillales	Lactobacillaceae	Lactobacillus	0.000222592	0.551919425
Firmicutes	Clostridia	Clostridiales	Eubacteriaceae	Eubacterium	0.077787779	0.282111008
Firmicutes	Clostridia	Clostridiales	Lachnospiraceae	Blautia	0.031229478	0.34112605
Firmicutes	Clostridia	Clostridiales	Lachnospiraceae	Butyrivibrio	0.189857936	0.211641113
Firmicutes	Clostridia	Clostridiales	Lachnospiraceae	Dorea	0.002656691	0.462516629
Firmicutes	Clostridia	Clostridiales	Oscillospiraceae	Oscillibacter	0.001074512	0.498036551
Firmicutes	Clostridia	Clostridiales	Ruminococcaceae	Anaerotruncus	0.067526704	0.291980007
Firmicutes	Clostridia	Clostridiales	Ruminococcaceae	Subdoligranulum	0.016713528	0.376268523
Firmicutes	Negativicutes	Selenomonadales	Veillonellaceae	Mitsuokella	0.079316686	0.280729335
Proteobacteria	Betaproteobacteria	Burkholderiales	Burkholderiales_noname	Burkholderiales_noname	0.755206703	0.050878373
Proteobacteria	Betaproteobacteria	Burkholderiales	Sutterellaceae	Parasutterella	0.231965492	0.193336483
Proteobacteria	Gammaproteobacteria	Enterobacteriales	Enterobacteriaceae	Enterobacter	0.001327494	-0.490072975
Proteobacteria	Gammaproteobacteria	Enterobacteriales	Enterobacteriaceae	Escherichia	0.006811505	-0.421099758
Verrucomicrobia	Verrucomicrobiae	Verrucomicrobiales	Verrucomicrobiaceae	Akkermansia	0.468353503	-0.117636023

Supplementary Table 2

	Sex (M/F)	Age (yr)	Diagnosis	**Medications past 30 days** (ND = no data)	Fecal calprotectin (ug/g feces)*
High inflammation	M	35	UC, pan-colitis	ferrous sulfate, amlodipine, prednisone	3975.1
	M	54	UC	prednisone, azathioprine, amlodipine besylate, furosemide, metoprolol	2870.5
	M	52	UC, pan-colitis	acetaminophen, balsalazide, enoxaparin, famotidine, levothyroxine, melatonin, methylprenisolone IV, ondansetron, tamsulosin, tramadol	1085.3
	M	38	UC**, pan-colitis	None	1005.9
	M	18	UC	melatonin	769.4
	F	35	UC	dicyclomine, mesalamine, multivitamin	552.0
Low inflammation	F	34	UC	NuvaRing, ranitidine, ibuprofen, Colace	39.9
	M	48	UC	metformin, Harvoni, insulin, NovoLog, lisinopril, citaprolam, olanzapine, ReVia, trazodone	8.9
	M	38	UC**	mesalamine, prednisone	7.2
	M	46	UC	nystatin, albuterol, amlodipine, atorvastatin, azathioprine, cholecalciferol, folic acid, hydrochlorothiazide, losartan, metoprolol tartrate, montelukast, omeprazole, ondansetron, prazosin, prednisone, quetiapine, tizanidine, allopurinol	7.4
	F	72	UC	ND	6.2
	F	43	UC	Solu-Medrol, morphine, Zoloft, gabapentin, pantoprazole, trazodone, ondansetron, Asacol	4.4
	F	38	UC	betamethasone dipropionate, clonazepam, fluticasone propionate, ibuprofen, sertraline	3.2
	F	76	UC	azathioprine, adalimumab	2.2
No inflammation	M	56	Healthy	ND	21.6
	F	18	Healthy	pantoprazole, Apri	19.3
	F	21	Healthy	None	7.1
	M	58	Healthy	None	6.6
	F	50	Healthy	Zoloft, clonazepam	6.6
	F	61	Healthy	metoprolol, naproxen, vitamin d	5.2
	M	37	Healthy	Symbicort, trazodone, albuterol inhaler, fluticasone inhaler	4.4
	F	62	Healthy	lisinopril, glipizide, aspirin, atorvastatin	2.5
	M	37	Healthy	Zoloft	2.4
	F	72	Healthy	ND	2.2
	F	40	Healthy	oral contraception	1.7
	F	27	Healthy	ND	1.3
	M	26	Healthy	ND	1.3
	F	64	Healthy	ND	0.9

*Fecal calprotectin determined by ELISA (Buhlmann)

**Same patient, two separate clinic visits
